# Extranodal Rosai-Dorfman Disease Involving the Left Atrium: Cardiac MRI, CT, and PET Scan Findings

**DOI:** 10.1155/2015/753160

**Published:** 2015-06-09

**Authors:** Vistasp J. Daruwalla, Keyur Parekh, Hassan Tahir, Jeremy D. Collins, James Carr

**Affiliations:** ^1^Conemaugh Memorial Hospital/Temple University, 1199 Mckinley Avenue, Johnstown, PA 15905, USA; ^2^Department of Cardiovascular Radiology, Northwestern University Feinberg School of Medicine, Chicago, IL, USA

## Abstract

Rosai-Dorfman disease (RDD) is a rare entity that usually involves the lymph nodes but extranodal involvements have been seen in numerous cases, although RDD with cardiovascular involvement is extremely rare. We describe a case of a young male who presented with intermittent palpitations and was found to have a left atrium mass. Our case not only emphasizes the rarity of the above lesion but also highlights the importance of modern-day imaging like computed tomography, Cardiac Magnetic Resonance Imaging (CMRI), and PET scan in characterizing such nonspecific lesions and directing appropriate line of treatment. RDD should be considered as one of the differentials even for isolated cardiac lesions.

## 1. Background

Rosai-Dorfman disease (RDD) is a rare, benign histiocytic proliferative disorder with massive cervical lymphadenopathy as the most common clinical presentation. Various extranodal lesions of RDD have been reported in the literature but involvement of the cardiac system is rare. The etiology of RDD is unknown and confirmation of the diagnosis still relies on immunohistochemistry analysis. We report a case of a young male presenting with intermittent palpitations and demonstrating a mass in the left atrium on imaging. Imaging like cardiac MRI, CT, and PET scan has proven to be immensely valuable in outlining such nonspecific lesions.

## 2. Case Presentation

**Figure 1:**
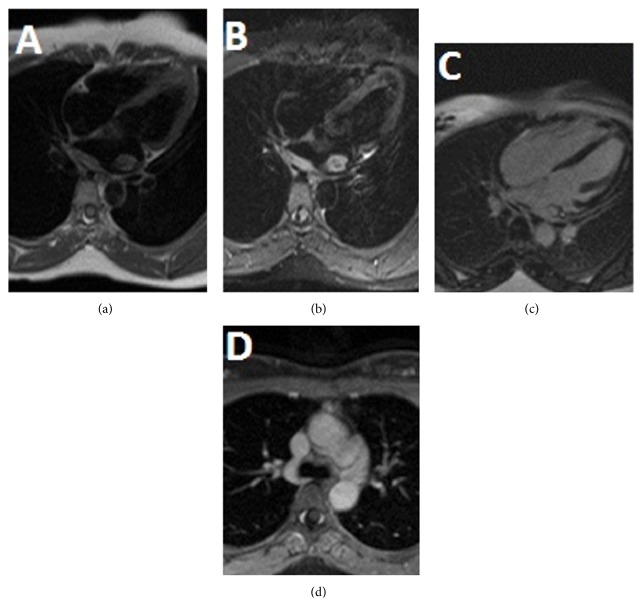
Cardiac MR (CMR) with and without contrast. T1 weighted dark blood image on 4-chamber view showed an isointense mass in left atrium with wall thickening along its posterior wall (a). Infiltrative nature of mass is noted in the form of hyperintense thickening of posterior wall of left atrium on T2 weighted dark blood image on 4-chamber view (b). Central T2 hypointensity, which is a common finding in RDD, can be seen in our case (c). Homogenous postcontrast enhancement is seen on delayed postcontrast 4-chamber view. Mediastinal lymphadenopathy with similar signal intensity as cardiac mass is seen on fat-suppressed postcontrast axial image (d). Lymphadenopathy is more marked in aortopulmonary window station.

**Figure 2:**
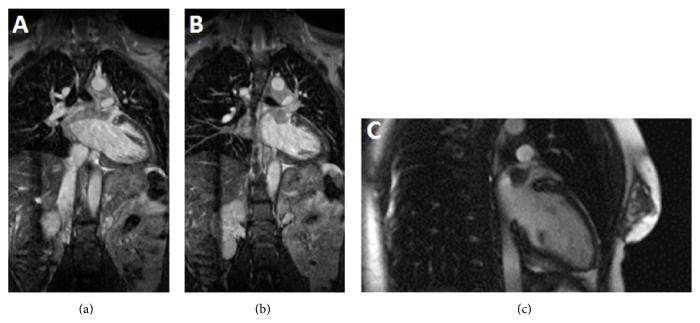
CMR—steady-state-free precession. Left atrial mass with infiltration of wall of left atrium in the form of wall thickening and postcontrast enhancement is seen on coronal steady-state-free precession images (a and b). Also seen is soft tissue mass in mediastinum with signal characteristics similar to left atrial mass. Precontrast 2-chamber view shows mass appearing isointense to left ventricular myocardium (c).

A 27-year-old African American male without any significant past medical history presented with intermittent palpitations and left ventricular hypertrophy on electrocardiogram (ECG). Two-dimensional (2D) transthoracic echocardiography showed an echodense mass in the left atrium. Further evaluation with Cardiac Magnetic Resonance Imaging (CMRI) demonstrated a heterogeneous broad base mass arising from the posterior superior wall and roof of the left atrium. The mass was located approximately at the expected location of coumadin ridge and measured 1.9 × 1.5 cm and demonstrated mild postcontrast enhancement (Figure 2). It arises from a diffusely thickened superior posterior wall and atrial roof but did not obstruct the pulmonary venous drainage at this time. Mediastinal lymphadenopathy with similar signal intensity as cardiac mass was noted on fat-suppressed postcontrast axial images (Figure 1). Lymphadenopathy was more marked in aortopulmonary region.

**Figure 3:**
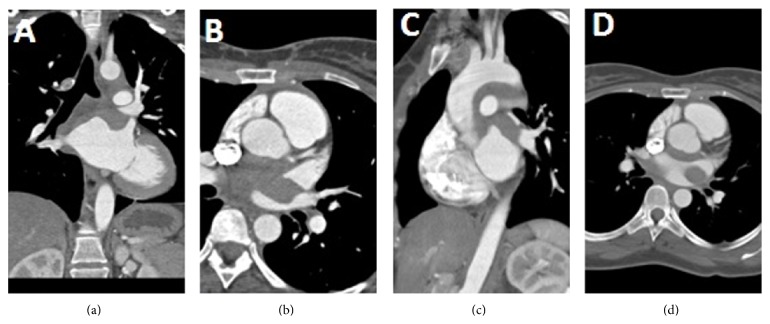
Prospectively ECG gated CT chest with contrast. Hypodense mass in left atrium is well demonstrated on coronal reconstructed images (a). CT scan helps in assessment of any possible compression of adjacent structures by mass lesion. Narrowing of left superior pulmonary vein is seen on axial image (b and d). Sagittal reconstructed image shows hypodense soft tissue thickening along the undersurface of arch of aorta that represents mediastinal lymphadenopathy (c).

Patient was further evaluated with Computed Tomography Angiography (CTA) of chest for better anatomical evaluation of the mass, which confirmed a cardiac region mass which is difficult to distinguish from the mediastinum and is located at the roof and posterior superior wall of the left atrium similar to the description on the CMRI (Figure 3). The mass demonstrated an infiltrative appearance and may actually originate in the mediastinum, exerting mass effect on to the left atrium. Based on findings of MR and CT, possibility of lymphoproliferative disorder was the primary consideration. PET scan was done to further validate the diagnosis.

**Figure 4:**
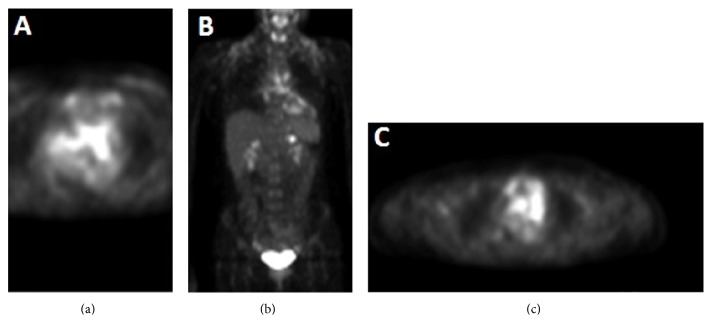
PET scan. Avid uptake of Fluorodeoxyglucose (FDG) in region of mass is seen in left atrium (a). Uptake is also seen involving mediastinal nodes. Coronal image shows uptake in cardiac mass and mediastinal nodes (b). Focal area of uptake was noted near upper pole of left kidney (c).

^18^F-fluorodeoxyglucose positron emission tomography has proven to be a valuable imaging technique for distinguishing neoplastic lesions from benign lesions and evaluating the extent and processes of the disease. It not only demonstrates the complete staging of the disease but also can provide functional information about the disease activity to guide biopsy. FDG PET imaging in our patient demonstrates foci of increased uptake in the mediastinum and right perihilar region, corresponding to soft tissue findings on the previous CT study (Figure 4). Focal area of uptake was noted near upper pole of left kidney, but, on further imaging by CT and MR of abdomen, no other definite lesion was identified. No other areas of uptake were noted on PET scan. PET scan is also a sensitive indicator for early prediction of treatment response in RDD.

**Figure 5:**
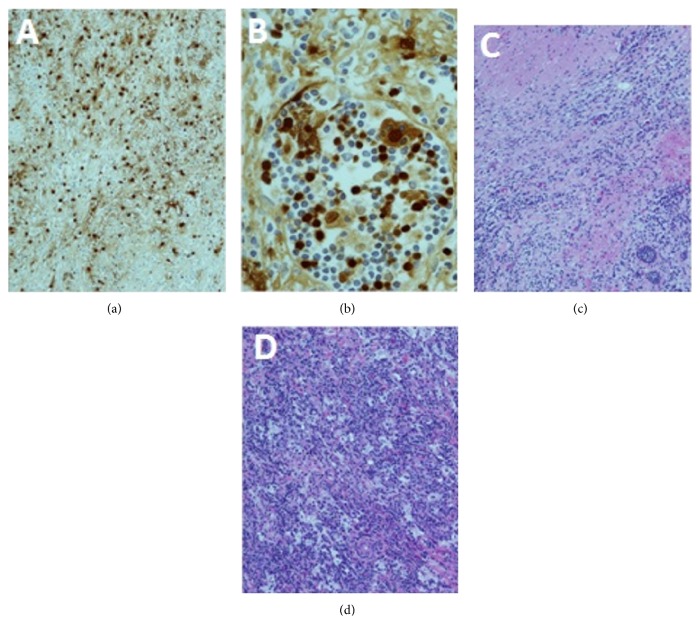
Immunohistopathology. Section through the left atrial mass shows histiocytes that are immunoreactive to S100 protein immunostaining (a). Emperipolesis is engulfment of lymphocytes and erythrocytes by histiocytes that is considered diagnostic of RDD. Emperipolesis is noted in section from left atrial mass on hematoxylin and eosin-stained sections (b). The histiocytes and lymphoplasmacytic cells infiltrating the myocardium of the left atrium are seen (c). Section from the left atrium also demonstrates fibrosis and large histiocytes in sheets that are accompanied by numerous plasma cells and small mature lymphocytes mass on hematoxylin and eosin-stained section (d).

Thoracoscopic-guided biopsy was inconclusive. Imaging done six months later showed no increase in size of mass lesion or mediastinal lymphadenopathy. Decision to surgically excise the mass was done. Left atrial mass demonstrated histiocytes which were immunoreactive to S100. Emperipolesis is engulfment of lymphocytes and erythrocytes by histiocytes which is considered diagnostic of RDD which was noted in the section from left atrial mass (Figure 5). The left atrial mass showed histiocytes and lymphoplasmacytic cells infiltrates with fibrosis and numerous plasma cells. Patient was discharged home and will continue follow-up with annual CMRI.

## 3. Discussion

RDD is also known as sinus histiocytosis with massive lymphadenopathy and is rare benign, self-limiting histiocytic proliferative disorder. The etiology is unknown; however the role of human herpes virus 6 and Epstein-Barr virus is suspected. There are approximately 1000 cases reported with a male predominance of 4 : 1 [1]. The typical age at presentation is the 2nd to 3rd decade. Fever, weight loss, and night sweats are common presenting symptoms, along with cervical lymphadenopathy on physical examination. There is a reported association between Rosai-Dorfman disease and sickle cell anemia. Many cases, mostly with nodal disease, usually resolve spontaneously. Complications are more commonly seen with extranodal disease due to local lymphadenopathy mass effect. The extranodal presentation in RDD occurs in about 30% to 40% of cases [2].

The head and neck are the most common sites for extranodal involvement with the nasal septum and parotid glands being the most commonly reported sites. Extranodal involvement has also been reported in the skin, orbits, salivary glands, bone, central nervous system, kidneys, and testes. Bony lesions are mostly lytic in nature; renal involvement is usually asymptomatic and presents with renal failure in the progressive stage of the disease. CNS involvement is in the form of dural-based lesions. Patients with involvement of extranodal sites tend have a fulminant course [3]. Unfortunately, there are no effective treatments for RDD. Various immunosuppressant and chemotherapeutic agents have been tried with limited success. Surgical removal is often required and complete resection generally results in cure. Radiotherapy can be applied to lesions, which are difficult or impossible to remove surgically. Our patient is being closely monitored with annual CMRIs to develop future treatment plans, based on further alterations in the lesion.

The differential diagnoses of a cardiac mass with microscopic features similar to those of RDD include Langerhans cell histiocytosis (in which the cells are positive for both S-100 protein and CD1a), an inflammatory myofibroblastic tumor (which has a background proliferation of spindle cells associated with an infiltrate of mononuclear inflammatory cells and ALK-1 positivity), metastatic malignant melanoma (in which cells are positive for Melan-A), Hodgkin's disease (which shows characteristic Reed-Sternberg cells and positivity for CD15 and CD30), and fungal or mycobacterial infections (which are validated by positive staining with GMS, PAS, and acid-fast stain) [4]. Literature review demonstrated three cases of right atrial involvement in RDD presenting with chest pain and hypotension or as an incidental finding, a relatively asymptomatic involvement of the left atrium as in our case has not been noted [5]. A case reported with epicardial involvement demonstrated a poor prognosis, resulting in patient's death [6]. The prognosis of atrial RDD remains unclear because cardiovascular system involvement is extremely uncommon.

Cardiac involvement in RDD is a rare occurrence and can mimic sarcoma, granulomatous disease, lymphoma, or benign atrial myxoma. Difficulty in evaluation of a cardiac mass by imaging is a frequently encountered scenario. Computed tomography (CT) helps us understand anatomical extent while magnetic resonance (MR) imaging and positron emission tomography (PET) scan can analyze morphology and metabolic features of a mass. Imaging helps in better characterization of a cardiac mass and also plays a pivotal role in assessment of its complications by demonstrating involvement of adjacent structures and mass effect or looking for a potential source of thrombus. Many pertinent clinical questions determining further management can be very well answered by appropriate imaging. Hence we illustrate the CT, MR, and PET findings of RDD presenting as a left atrial mass.

## 4. Conclusion

Rosai-Dorfman disease (sinus histiocytosis with massive lymphadenopathy) is a rare entity with extremely uncommon involvement of the heart as an extranodal lesion. Imaging plays an important role in the diagnosis and management of lymphoproliferative disorders like Rosai-Dorfman disease which commonly present as nonspecific lesions. Although immunohistopathology remains as the main stay for confirmation of the disease, CT, MRI, and PET scan imaging are extremely useful in early detection and localization of the mass. PET scan is also a sensitive indicator for early prediction of treatment response in RDD. Through this case, we not only stress the importance of maintaining a high index of imaging suspicion for RDD but also delineate the significance of imaging in management and follow-up of such cases.
